# Recovering Context in Psychiatry: What Contextual Analysis of Service Users' Narratives Can Teach About Recovery Support

**DOI:** 10.3389/fpsyt.2021.773856

**Published:** 2021-12-20

**Authors:** Nienke van Sambeek, Andries Baart, Gaston Franssen, Stefan van Geelen, Floortje Scheepers

**Affiliations:** ^1^Department of Psychiatry, University Medical Center Utrecht, Utrecht, Netherlands; ^2^Department of Humanities, Optentia Research Unit Focus Area, North-West University, Vanderbijlpark, South Africa; ^3^Department of Dutch Literary Studies, Faculty of Humanities, University of Amsterdam, Amsterdam, Netherlands; ^4^Education Center, University Medical Center, Utrecht, Netherlands

**Keywords:** mental health recovery, lived experience, context, narrative characteristics, qualitative research

## Abstract

**Aim:** Enhancement of recovery-oriented care in psychiatry requires insight into the personal meaning and context of recovery. The Psychiatry Story Bank is a narrative project, designed to meet this need, by collecting, sharing and studying the narratives of service-users in psychiatry. Our study was aimed at expanding insight into personal recovery through contextual analysis of these first-person narratives.

**Methods:** We analyzed 25 narratives, as collected through research interviews. To capture the storied context on both a personal, interpersonal and ideological level we combined several forms of qualitative analysis. A total of 15 narrative characteristics were mapped and compared.

**Results:** Through comparative analysis we identified four narratives genres in our sample: Lamentation (narratives about social loss), Reconstruction (narratives about the impact of psychosis), Accusation (narratives about injustice in care), and Travelogue (narratives about identity transformation). Each genre provides insight into context-bound difficulties and openings for recovery and recovery-support.

**Conclusion:** A contextual approach to studying personal recovery offers insights that can help attune recovery support in psychiatry. Important clues for recovery support can be found in people's narrated core struggle and the associated desire to be recognized in a particular way. Our results also indicate that familiarity with different ways of understanding mental distress, can help people to express and reframe their struggles and desires in a helpful way, thereby facilitating recognition.

## Introduction

Enhancing personal recovery has become central to mental health care reforms across the Western world (1, 2). Grounded in the experiential knowledge of psychiatric service-users, personal recovery has been characterized as a deeply personal process (3, 4) that involves processes of connectedness, hope and optimism about the future, identity, meaning in life and empowerment (5)^1^. This conceptualization can be contrasted with the cure -oriented concept of “recovery as remission of illness”– or clinical recovery- that has dominated psychiatry for the last decades (7). Research has demonstrated that personal and clinical recovery are not necessarily associated (8–11). As a consequence, symptom focused treatment is not likely to enhance personal recovery without additional support (11).

In order to sustain such recovery support, an amplification of resources and methods in psychiatric research is needed. Numerical and general evidence on recovery, as generated by dominant quantitative research methods, is unable to gain insight into the context and meaning of personal recovery. To understand what is meaningful and relevant to a person, requires attention for the subjective experience of recovery, with reference to the persons' life history and socio-cultural context [(12), 234]: Traditionally, the recovery approach has been intertwined with narrative research, as the philosophical underpinnings of these “paradigms” strongly align (13). Moreover, narrative research allows for the synthesis of personal stories, enabling them to be considered a source of evidence (14). However, the study of narrative has developed largely outside of psychiatry (15). Over the last decades, various research-practitioners have made a case for qualitative, humanities-based inquiry of patients' narratives in medicine. They have argued that listening to the stories of people with lived experience is indispensable to come to a deeper understanding of recovery and to advance person-oriented care (15–18).

The present paper presents a study that was designed to obtain contextual understanding of personal recovery to inform psychiatric practice. For this aim we scrutinized the narratives of service-users as collected in the “Psychiatry Story Bank.” Our study departs from a social-constructivist philosophy. We approach the narratives not as neutral reflections of the tellers' world, identities and social relationships, but as playing an active role in creating them (19). Their stories are shaped by the social context, but their stories, too, shape that context. Hence, we assume that the way people construct meaning in their narratives has real life impact on their recovery (15, 20). This philosophy aligns with widely used concepts of personal recovery as a process of transformation of meaning making (3, 4) and narrative as one of the key instruments to enact such transformation (21, 22).

Previous qualitative research on personal recovery has focused mainly on similarities in service-users accounts, identifying key processes (5, 23, 24), phases (25) and hindering and facilitating factors (24, 26). Based on the initial exploration of our narrative data, we concluded that the unique context they offer, holds out the prospect of adding valuable insight into the lived experience of recovery, as the “storied context” illuminates differences in what is of importance to whom, and what assumptions and experiences tellers rely on. Although a focus on understanding phenomena in context, is central to qualitative research in general, and narrative research in particular (27, 28), translating it into the process of data-analysis is a challenging and under-addressed issue (28, 29). In this study, we build on two important leads for analyzing storied context from the narrative literature. We will shortly discuss these leads and their significance for recovery research.

The first lead is found in the idea that a narrative has an internal, or hermeneutic context. In order to preserve this context, narrative data should be studied as a holistic unit. This “holistic principle” is considered to be a central and distinguishing principle of narrative analysis (30–32). It maintains that various aspects of individual experiences are intertwined in the narrative, and therefore cannot be understood separately (13). Analysis, then, should be directed at preserving the personal context and coherence of narrative accounts. In the case of recovery narratives, holism is argued to be urgently needed because it enables the appreciation of people living with mental distress as multifaceted persons with experiences beyond illness (13) and for the embedding of their experiences into a narrated life history (33).

The second lead entails the idea that the individual narrative is related to and structured by an external, or socio-cultural context that needs to be explicitly addressed in analysis as well (20, 31, 33–36). As Murray points out: “In telling his or her story the narrator makes use of socially embedded language. It is not fully the narrator's story: its structure is conditioned by both the immediate presence of others and the dominant plot in society” [(37), 344]. Thus, he encourages researchers to move beyond analysis on the personal level of the narrative, to include and connect interpersonal and ideological levels of context as well.

The interpersonal context, refers to the influence of the listener or imagined audience that influences how narratives are shaped (37). People enact identities, justify actions (38) and seek recognition (17) through the stories they tell. Thus, analyzing narratives as “performances” with rhetorical properties, can provide insight into how people want to be understood (35, 39–41), thereby respecting the narrators' desires and intentions (33, 42).

The ideological context refers to the embedding of individual narratives in culturally shared stories, or meta-narratives. These meta-narratives comprise dominant plots about illness, normality and the meaning of suffering (37). For example, the dominant medical meta-narrative in the Western world has been characterized by sociologist Arthur. W. Frank as the “restitution narrative,” in which return to health and normality is desired and symptoms have to be controlled through professional intervention. As Frank demonstrates, the reconstruction narrative falls short in the case of chronic illness and can undermine ill peoples' capacity to become the hero of their own story (17). In the case of mental health recovery research, it is important to analyze and explicate these meta-narratives and their consequences, in order to facilitate the construction of destigmatizing counter-narratives (13, 37, 43, 44). In fact, the creation of normalizing and self-authored narratives that encourage people to rediscover their selves beyond illness have been central to the empowerment of service users (45).

Although the importance of holistic analysis of both the personal, interpersonal and ideological context of narratives in recovery research may seem evident in theoretical elaborations, this importance is not clearly reflected in prevailing research practice. Reviewing narrative studies on mental health recovery, Spector-Mersel and Knaifel (13) identified that the holistic principle was often disregarded in the process of data-analysis. Other scholars have observed that researchers often neglect the socio-cultural context of narratives (35), or exclusively focus on either the personal or social dimensions of the telling (37, 46).

In our research we attempt to address these forms of de-contextualization, in order to come to a better understanding of personal recovery. Based on the literature and reflective engagement with the narratives under study, we put together an analytical framework that facilitates contextualization of personal recovery. Our central research question was: What can we learn about personal recovery and recovery support through analysis of the storied context that first-person narratives offer? Below we will first outline the context and methodology of our research project and explicate how we elaborated the idea of personal, interpersonal and ideological context into an analytical framework for inquiring psychiatric narratives. Subsequently, the outcomes and merits of a contextual approach for improving recovery support in psychiatry will be discussed.

## Materials and Methods

### Setting

This study is part of the “Psychiatry Story Bank,” a project initiated by the department of psychiatry of the University Medical Center of Utrecht (The Netherlands). In this project, we invite service-users, their loved-ones and (informal) caregivers to share their stories through an open interview. The aim of the project is to improve psychiatric care and recovery support, through the study of personal stories. This initial study focused on the stories of the subgroup of service-users to inquire personal recovery.

### Sampling and Participants

Participants in this study were people that made use of psychiatric services in the Netherlands. Thus, enrollment was not limited to service-users of our own department. Apart from acute crisis, no exclusion criteria were formulated. In line with the latest clinical (47) and narrative (43, 44) insights, we took a trans-diagnostic stance in our study. Initially, participants enrolled on the basis of convenience sampling. People could sign up through our project website, advertised on (social) media, at conferences, and by word-of-mouth. As we determined a selection bias toward highly educated, employed and native Dutch participants, we adapted our recruitment strategies to maximize variation (48). With the help of caregivers and community peer-workers in less advantaged neighborhoods, we reached out for groups that were under-represented. In the cyclic process of sampling and analysis, we noticed that variety in demographic characteristics elicited more narrative diversity as well. After 25 interviews, saturation was established (49). The characteristics of the final sample are displayed in Table 1.

**Table 1:** Characteristics of the participants (*n* = 25).

**Gender**	**Participants**	**Age**	**Participants**
	**(***n***)**		**(***n***)**
Male	11	20–40	5
Female	14	41–60	13
		61–80	7
**Country of origin**		**Employment**	
The Netherlands	20	Employed	10
Other (Western)	1	Unemployed/retired	8
Other (Non-western)	4	Volunteer work	7
**Highest education**		**Income**	
Primary school	3	Above average	5
Secondary school	4	Below average	7
Vocational education	3	Minimum income	13
Professional education	8		
University	7		
**Partnership status**		**Children**	
Partner	10	Yes	13
No partner	15	No	12
**Living situation**		**Care history**	
Independent	23	Multiple hospitalizations	12
Assisted living	2	Single hospitalization	5
		Only outpatient care	8
**Diagnostic group**			
Mood disorders	10	Neurocognitive disorders	1
Personality disorders	7	Impulse control disorders	3
Psychotic disorders	7	Dissociative disorders	2
Developmental disorders	3	Eating disorders	1
Trauma and stress related disorders	10		

^*^Based on self-reported diagnoses, clustered in diagnostic groups as suggested by Delespaul (50). Most people reported diagnoses in more than one group.

### Data-Collection and Procedure

After signing up, people were first contacted by phone to be informed about the aim and process of participation. Additionally, they received an information letter with details about the study and the way their personal data would be treated. When people gave their consent to participate, the interview was planned and conducted at a place of the participants choosing, often resulting in home visits. All participants provided their written informed consent. Interviews lasted 70 min on average. They were all audio taped, transcribed literately and stored anonymously in a secured database

Interviews were administered by the first author and an interview pool of junior mental health-care professionals between May 2018 and July 2019. We combined narrative-and semi-structured interview techniques. The open-ended topic guide to inquire personal recovery (see Appendix I) was used in a flexible way. Participants were encouraged to guide the direction of the interview with the opening question: “What story would you like to share?” Consequently, interviewers were trained to minimize interference with the narrators' conversational flow, only intercalating into the telling to create more depth and fill in gaps after receiving clear signs that the interviewee had finished their story (51). All interviewers were trained and supervised by the first author and project team during the course of data collection. After their first unsupervised interview, the quality of the interview was systematically evaluated, and desirable techniques and pitfalls were discussed. Acknowledging the interview as a co-construction (33), feedback was also directed at enhancing awareness of the interviewers-often unconscious- narrative preferences and discomforts (17).

In order to ensure that participation in our study would be a recovery supportive experience, interviewers were sensitized in terms of recovery support by an expert-by-experience from our project team. Additionally, all research participants were offered the opportunity to have an edited and anonymized summary of their narrative published on our online platform^2^. The platform was designed to facilitate the personal and social benefits of story sharing for both the teller and the recipients (44). Participants were invited to fill out an evaluation form afterwards. This feedback was used to improve our practices.

### Analysis

Our analysis was guided by the principles of a holistic, interpretative strategy. Most importantly, this approach requires to treat the narrative as a whole, to regard both form, content, and contextual embeddedness, and to study the data from an interdisciplinary and multidimensional lens (32). Our focus in analysis was to illuminate and conserve the storied context that narratives offer, as a means to contextualize recovery. We approached the narratives as momentary, subjective evocations and evaluations of the personal and social contexts that shape their narratives. Analyzing their stories from this perspective, then, allows us to “recover” those contexts. In line with the suggestion of Murray (37)^3^ and other authors, we distinguished between the personal, interpersonal and ideological level of context in our analysis. Because most of the common methods in qualitative research do not cross the “categorical binary of individual and society” [(46), 190] we needed to combine several methods of analysis for this purpose. The analytical framework that is presented in Table 2 is the result of a dynamic and abductive process. Thus, insights that developed while reading the narratives and exploring different forms of analysis, encouraged further reading, which in turn refined our analysis, and so on. The analyzed characteristics were selected on the bases of two criteria; (1) the (related) characteristics provide insight in the narrative as a whole; (2) the characteristics differentiates between narratives, thus providing insight in the specific context. For an extensive overview of the literature and methods that underlie the analysis of narrative characteristics, we invite the interested reader to consult Appendix II.

**Table 2:** Analytical framework.

**Level of analysis**	**Personal**	**Interpersonal**	**Ideological**
Subject of analysis	Storyline/content	Form/rhetoric	Discourse/language
Analyzed characteristics	***Subject*** What is the narrative mainly about? ***Themes*** What are the central issues brought forward in the narrative? ***Life story*** How can the narrated life-story be characterized? ***Struggles*** Which urgent personal struggles are expressed in de narrative? ***Resolving*** How is the core-struggle overcome or dealt with?	***Purpose*** What is the purpose of the telling? ***Audience*** Who is the intended recipient of the telling? ***Emotional tone*** What is the prevailing expressed emotion in the telling? ***Structure*** How is the telling structured? ***Appeal*** How does the narrator want to be recognized by the audience?	***MD construction*** How does the narrator construct his/her understanding of mental distress in language? ***MD framework(s)*** Which framework(s) of mental distress are dominant in the narrative? ***Related identity*** Which identity is made possible by the narrator's preferential framework of mental distress? ***Related function*** What is gained by using this framework? ***Related responsibility*** What are the consequences of the framework in terms of actions to be undertaken for recovery?

Analysis was carried out by the first author, in interaction with a multidisciplinary research team. The first author is a former mental health practitioner with experiential knowledge, thus uniting different perspectives. The research line was determined by our project team, including both mental health practitioners and experts-by experience. Data analysis lasted for a year, including extensive reading of the interviews and interview memos. The second author closely supervised the process of methodology development. In an early stage, three cross-reading sessions between the first and third author were held to compare and amplify the reading of the narratives. After the initial analysis, we identified the differences and similarities across cases in a comparative analysis (52). Shared narrative patterns were further analyzed, contrasting cases and defining prototypical and negative cases. This process resulted in the description of narrative genres that were validated by the third and fifth author. Revision of all case summaries resulted in a 92 percent interrater correspondence on the primary genre.

Additional to researcher triangulation, we engaged in member checking to augment the credibility of our analysis. Since our research is aimed at transforming mental-health practice, we broadened the notion of member-checking by including intended users of the research, practicing so-called “audience-validation” (53). In total, we organized three feedback sessions of 90 min, with respectively a lived-experience panel (*N* = 5) and two practitioner panels (*N* = 8). In these sessions, our provisional findings were submitted and discussed to verify their credibility, value and effect. From the sessions with practitioners, we learned that genres were found credible, but carried the risk for reification. Experts by experience further encouraged a contextual approach to understanding recovery, but warned for an overly neat and systemized framing of the process of recovery, that they experienced as rather chaotic, asynchronous and ambiguous. Another point of critique was the use of professional health care terminology in the description of the results. We revised our results description, incorporating these points of critique.

## Results

In order to come to a contextual understanding of recovery in psychiatry, we analyzed the narratives of 25 service-users. Our framework for analysis was designed to capture both the personal, interpersonal and ideological context of the narratives. Comparative analysis led to the distinction of four genres, which we named Lamentation, Accusation, Reconstruction and Travelogue. We understand these narrative genres as forms of rhetorical action that are specific to the research setting and interaction, as opposed to static categorizations (36, 54, 55). A synthesis of the characteristics of each genre and context is displayed in Tables 3–5.

**Table 3:** Syntheses of results on the personal level.

***Genre***	***Subject***	***Main themes***	***Life story***	***Core struggle***	***Resolving***
Lamentation	Mental distress	Loss of familiarity Self-loss Shame Social support	Marginalization	Struggling with social decline	Finding socially accepted ways to express pain
Reconstruction	Mental distress	Anxiety Alienation from reality Hospitalization Career disruption	Over-demand	Struggling with meaning	Meaning making through occupation
Accusation	Care	Dependency Stigma Power- inequality System barriers	Deviation	Struggling with rejection	Finding a committed caregiver
Travelogue	Recovery	Disturbed childhood Emotional expression Self-insight Empowerment	Adaptation	Struggling with neglected needs	Surrendering to repressed pain

**Table 4:** Synthesis of results on the interpersonal level.

***Genre***	***Purpose***	***Audience***	***Tone***	***Structuring***	***Appeal***
Lamentation	Share grief safely	Interviewer	Sorrowful	Associative	Recognition of dignity
Reconstruction	Order experiences	Self	Wondering	Chronological	Recognition of capability
Accusation	Convince Provoke change	Professionals	Resentful	Argumentative	Recognition of humanity
Travelogue	Inspire Belong	Peers	Reflective	Plot driven	Recognition of sensitivity

**Table 5:** Synthesis of results on the ideological level.

***Genre***	***Mental distress construction***	***Mental distress*** ***Framework(s)***	***Related identity***	***Related function***	***Related responsibility***
Lamentation	Mental distress as weakness	Taboo framework	Tough person	Protects dignity	Restoring normality
Reconstruction	Mental distress as social isolation	Medical and participation framework	Recovering client Person with psychiatric history	Facilitates acceptance of assistance Offers future perspective	Symptom- management Avoidance of distress Occupational engagement
Accusation	Mental distress as a necessity for care	Social justice and humanistic frameworks	Injustice fighter Human being	Reduces feelings of powerlessness Commits others to care	Not giving up on life Fighting to get the right care
Travelogue	Mental distress as disconnected self	Psychotherapy, recovery and spiritual frameworks	Vulnerable person Expert by experience	Legitimates and values vulnerability	Being connected and open to self and others

The overview of characteristics is meant to provide insight into the differences between, and relations within the genres. However, in order respect and demonstrate the holistic character of the narratives, we will illustrate the outcomes by discussing four narratives that represent the different genres. Below, we will set out what these narratives can teach us about recovery, and illustrate how contextual analysis helped to come to these insights.

### Lamentation (*N* = 3)

This genre was scarce in our sample, and particularly represented by people that were recruited in community centers in less advantaged neighborhood. The name refers to the grief that is expressed by the tellers.

Through a contextual analysis of these narratives, it became apparent how stigma can deepen the experience of loss brought about by mental distress. We will illustrate this with the story of Rana, a 67-year-old widow and mother, with a non-western migration background. Rana lives independently and has a volunteer job. She signed up after meeting the interviewer at her community center.

#### Interpersonal Context

Rana does not explicitly refer to a purpose or audience she has in mind. Her telling is directed at the interviewer, whom she welcomes as a new friend. Rana tells in a dramatic, and associative way, moving back and forth between scattered memories. She indicates that her loved-ones lost patience with her sorrow. Participating in our study, then, seems an opportunity for emotional support to her: An encounter to share her grief without risks. We identified the appeal of her story as a demand for recognition of both her pain and dignity. She seems to implicitly ask “Am I still worthy, considering everything I have lost?”

#### Personal Context

Rana's narrative evolves around experiences of mental distress and the impact it has had on her social life. She tells of how she “lost her way” after the death of her husband, and discloses how she used to dwell the streets, feeling lonely and anxious. Other parts of her narrative involve references to her hospitalization. Through analysis of the personal context, we learn that Rana's mourning exceeds the loss of her husband. Loss of social support, normality and identity are important themes of her narrative:

“*Every time I get into trouble, I see my deceased father in my dreams, and beg him: ‘Help me, I just want my normal life back', but this- I just can't manage (…) I want the old Rana back… She was fun, sociable. I mean I can still… that's what they say - But inside I am pain, sorrow, lots of sorrow.”*

Throughout her telling, Rana depicts her “old self” as an outgoing and tough woman. She illustrates how she used to enforce respect upon others, using her “mouth as a weapon”- an attitude that gains meaning in the light of her narrated life-history. Rana refers to the impact of growing up in the harsh environment of an immigrant camp. She relates the experience of witnessing domestic violence in her community as a child, to a decision to arm herself to keep her family save. She seems to perceive of her current troubles as a threat to her carefully built up social status and struggles with the social decline that her loss of “normality” entails. Between the lines, she sketches an image of various friend and family members that distanced from her when she lost her old ways:

“*They said, this is not how we know our mother, this isn't her. You know? Especially my grandchildren took it badly. Cause when I was there [in the institution], they didn't' want to visit their grandmother. Even though I asked them to. But they said: this is not our grandmother.”*

Although her hospitalization has been a shock to Rana, she also relates it to the “discovery of her creativity”. She tells of how her mentor “is worth gold to her,” encouraged her to involve in expressive therapy, and arranged a workshop for her after her dismissal. She indicates that art continues to be an important outlet for her sorrow and that her grandchildren have already claimed some of her works. Art, then, seems to offer a starting point for resolving her struggle with social decline and a possibility to regain her dignity.

#### Ideological Context

Rana's narrative draws heavily upon a stigmatizing framework. She conceptualizes mental distress as a form of weakness. Consequently, acknowledgment and disclosure of mental distress seems taboo to her. Moving on with life, and “being among normal people” is what Rana longs and strives for in recovery. She seems to protect her dignity by splitting: referring to her distressed self as “another lady.” Rana resents her children for administering her to a psychiatric ward, and dissents fiercely from her fellow service users by devaluating them as “crazy”, “dumb,” “smelly” and “pitiful.”

“*I do blame my children, you know. I mean, it had to- But I blame them for sending me there [the institution]. Of course they couldn't stay with their mother all the time. But then I ended up there (…). And I thought, what am I doing here between all those crazy people?”*

Rana seems to have limited access to other, more compassionate frameworks of mental distress. However, a framework of expressive therapy apparently offers her an opening to a new story: One that values the outlet of mental distress and offers socially respected ways to do so.

#### Conclusion

Through contextual analysis of Rana's narrative, we learned about the mutual enforcing relationship between her mental distress and loss experiences. We came to understand that mental distress poses a serious threat to Rana's social status and support system and nourishes her desire to restore normality. Rana's narrated life-story sheds light on the importance of strength, and the urgency to protect her dignity. However, understanding mental distress as a form of weakness has brought Rana to a deadlock in redefining who she is amidst mental distress, leaving her no possibilities but denial and rejection of who she has become. The opening for recovery in her story consists of the discovery of art as a means to express her pain. Art seems to offer her possibilities to reconnect with her loved ones and to elicit recognition for both her sorrow and dignity.

### Reconstruction (*N* = 5)

This genre was specific to people that lived through psychosis and mainly represented by men. Most of the narrators were encouraged to participate in the study by someone within their network. The name refers to the tellers' endeavor to recall and own past events.

Through contextual analysis of reconstruction narratives, it became apparent how making meaning of one's life is complicated by the specific experience of psychosis. We will illustrate this with the story of Robert, a 40 year old, single and native Dutch man. Robert is living independently and has a volunteer job. His peer worker encouraged him to participate in the project, after initial hesitation.

#### Interpersonal Context

On beforehand, Robert indicates he is “not usually occupied with events from the past” and that it might be hard for him to talk on his own initiative. The interview takes him considerable effort. Robert's telling seems self-directed, as he is very concentrated and turned inward. It appears as if he is ordering his past for the first time. In doing so, he depicts the long way he has come. Both the content and act of his telling seems to confirm his fragile, but growing sense of possibilities in life. Consequently, his telling was read as an appeal for recognition of his capability.

#### Personal Context

Robert's narrative is mainly about the experience and impact of mental distress on his life course. Lost future perspective, particularly in terms of a professional career, is a recurrent theme of his narrative. Robert tells how his problems -diagnosed as “disorganized schizophrenia” -prevented him from graduating high school, leaving him bereaved of his dream to study medicine. He also tells about the overwhelming anxiety and distrust he experienced during various psychotic episodes as well as the deep depression that followed after hospitalization. His difficulties to retrieve past events can be contrasted with his clear recalling of emotional states.

“*I can't clearly recall, but- I have this disorganized form- but, the fear, for being chased and such (…) There was a certain hesitation and insecurity, like is it really happening or not? Like a constant questioning if everything around me was true. So when I went out I was worried that someone would put something in my drink, that sort of thing. Then I had these panic attacks. Eh, like I couldn't hold it together anymore.”*

Throughout his telling, Robert expresses how frightening it has been to him, to be confronted with an abundance of ambiguous meaning. Robert reports how pharmacotherapy immediate relieved him from this burden. Although he struggled to bear the side-effects, and accept his reliance on it, he identifies medication as his most important helper.

With regard to his life-history, it is remarkable that Robert excludes his childhood from his telling. He also prefers not to go deeper into heavy-laden subjects, such as the harassment by his peers in high school -that preceded his first psychosis- and the disrupted relationship with his parents. He does indicate, however, that these events still provoke anxiety and hinder him in his social life. The main struggle we identified in Roberts' narrative is a struggle to find meaning, both in attributing meaning to distressful experiences and in envisioning an alternative future. It appears that Robert prefers to seek meaning in the present, rather than in the past. We learn that this preference is met in the rehabilitation program he currently enrolls in. In occupation, Robert seems to have found an important key in resolving his struggle with meaning.

#### Ideological Context

Ideologically, Robert's story aligns mostly with a medical framework, locating his condition in his head and talking about progress in terms of symptom containment. The effectiveness of medication apparently shaped Roberts' thinking:

“*During one hospitalization -about ten years ago or so- I got a different medicine. One that could potentially be dangerous. Something with thrombocytes I believe. But that, ehm, is tested every month and everything is okay. Those medicines really helped me, in combination with two other pills. So I am like double or triple protected. Well, I think that was a real good move. It really helped me. I finally found some peace, in my head.”*

A medical framework apparently helped Robert to accept assistance in tempering the highly distressful experience of psychosis. However, on an existential level, it left his struggle with meaning unaddressed. In contrary, a focus on stabilization seems to sustain his avoidance of situations that might provoke distress. By contrast, the recovery framework that was recently conveyed by his (peer) social workers seems to have encouraged Robert to envision himself as a person with possibilities in life and to explore those possibilities step by step. Although Robert is still struggling to find out what is feasible for him, he found a way of being the student he once imagined himself to be.

“*Through my volunteer job I noticed that I could still work on myself. I started reading about didactics, learning strategies and such.”*

“*I do feel the absence of a family, a relationship. But well… I feel like I have found a way of living with this [home] studying. I don't have to think about my purpose in life anymore. It has become a way of living. Like monks that meditate all day.”*

#### Conclusion

Through contextual analysis, we gained insight into Roberts' complex relationship with meaning-making. Making meaning of the past has become a frightful endeavor for him, as his very anguish is constituted by the ambiguous meaning he experienced in psychosis. Robert came to distrust his perceptions and shows reluctance to look back at distressful events. At the same time we learn that Robert struggled with the loss of his envisioned life. In the context of these challenges to make meaning of both past and future life, Robert finds satisfactory meaning by living in the present moment and keeping his mind focused, with the help of medication. For Robert, occupation turns out to be an important means to address his existential struggles, and to revision himself as a person with capabilities.

### Accusation (*N* = 8)

Stories of this genre were told by people with diverse backgrounds and diagnoses that enrolled on their own initiative. The name refers to the resentment over injustice that is expressed by the narrators.

Through a contextual analysis of these narratives, it became apparent how fighting others, may become a last resort to suppress experiences of powerlessness and demoralization. We will illustrate this with the story of Ida, 50 year old woman with a Western migration background. She lives independently and is self-employed. Ida signed up for an interview through our website.

#### Interpersonal Context

In the enrollment form, Ida introduces herself as someone who is considered both a successful businesswoman and a “confused person” in society. Aware of her acquired capabilities and resources, Ida feels responsible to speak not only for herself, but also for unheard others. Ida is explicit about the purpose and intended audience of her narrative: She wants to confront professionals and policy makers with the consequences of an over-specialized mental health care system that excludes people with severe mental illness from adequate treatment. Her narrative is a resentful, argumentative report of the injustice she has experienced in care. Her literal appeal is to be to be recognized “as a human being with normal emotional needs” and treated accordingly.

#### Personal Context

The subject of Ida's narrative is psychiatric care. Her narrative evolves around 25 years of medical encounters and her fight to get the right care. Stigma, power inequality and system barriers in care are central themes she addresses in her narrative. Ida sets out her experiences of refusal and maltreatment by mental health institutions. She recalls how she has been repeatedly deemed too complex and risky to treat, eventually hitting the bottom when she was registered as “damaged beyond recovery,” making her feel powerless, and deprived from opportunities to grow.

We identified a struggle with rejection as central to Ida's narrative. Although she does not foreground her life-history, she depicts a background of severe childhood trauma and disapproval. Ida continues to feel that people usually turn away from her, in response to her troubled behavior. Whenever she senses signs of rejection, she feels she needs to “get away from the unbearable,” resulting in dissociation and suicide attempts. The tragedy for Ida is that her expectation to find safety in care is not met. Instead she finds herself trapped in patterns of rejection again.

Resolving in Ida's narrative consists in finding a therapist willing to commit to her. She tells how she refused to give up hope after her final dismissal from the institutions and how she managed to find an independent psychiatrist with the help of her social network. In the extract below, she describes the healing experience of being in a safe therapeutically relationship:

“*It was very special to experience, through him [the therapist] that I'm not solely a monster, or that I am not a monster, but I perceive myself as a monster. And that I am a person who is doing things, and who is capable of having a reaction toward someone else. He really motivated me, with his support, to dare to reflect on myself. I knew… or there were moments I dared to understand that he was solidary and had the courage- That he was not afraid of me and would not break off the contact. He showed me over and over again, that he was not leaving.”*

#### Ideological Context

Ida conceptualizes mental distress as a necessity for care. Drawing on frameworks of attachment theory and ethics of care, she locates recovery exclusively within a healing, therapeutic relationship. These frameworks seem helpful to her, as they validate her feelings of dependency and urges others to care. However, she feels that the way she understands her own distress has been regularly neglected and silenced by professionals. She indicates that the initial framing of her troubles as “Borderline Personality Disorder” by medical professionals has been particularly damaging for her identity.

“*Borderline is a scary diagnosis (…) When you read that list, those nine points, then you are facing a monster. I find that difficult. And well, I find it is badly described, from my perspective- So well, I was shocked, that apparently, that was me (…) And I noticed that it elicits discrimination. With this diagnosis, you are constantly perceived as someone that manipulates, that's seeks attention. So the diagnosis wasn't helping, because caregivers find it difficult to connect to someone with a diagnosis of borderline. And they are right. But that doesn't mean it is impossible.”*

Despite her awareness and fight against stigma, it causes her trouble to liberate herself from the “monster” identity she apparently internalized. Adapting a framework of social justice, then, transforms Ida's personal struggles in political and legal action and seems an important means to counterbalance the powerlessness she experiences.

#### Conclusion

Through contextual analysis of Ida's narrative, we gained insight in her complicated voyage from powerlessness and demoralization, to growing empowerment. We came to a deeper understanding of Ida's desire to be recognized in her humanness. After a lifelong experience of deviation and rejection, her appeal is to be seen in her similarity to others. Although she tries hard to disentangle from the stigmatizing stories she has been caught up in most of her life, she feel powerless to change the way she is perceived and treated by others. Her encounters in- and exclusion from the psychiatric system enhances her feelings of powerlessness and demoralization. Accusation and legal justice, then, seem the only means left to her to gain some control: they confirm to her that she is a human being that deserves equal respect and rights as others. Intellectual empowerment apparently serves Ida to articulate her concerns and needs. However, it is the enactment of a humanistic and de-stigmatizing approach by others that she relies on.

### Travelogue (*N* = 9)

This genre was represented by people that enrolled on their own initiative, and had often shared their story previously in a peer-to-peer setting. Salient was that most of the tellers reported to be the child, sister or brother of a person suffering from mental distress. Tellers typically described their recovery as a journey of transformation.

Through a contextual analysis of these narratives, it became apparent how recognition of sensitivity can facilitate a sense of belonging. We will illustrate this with the story of John, a 53 year old, native Dutch man. He lives independently, together with his wife and children and is employed as a peer-work coordinator.

#### Interpersonal Context

John's narrative is a reflective and plot-driven account of lessons learned in the process of recovery. John perceives these insights as tools that can help himself as well as his peers. As John repeatedly stresses and legitimates his “brokenness,” his narrative reads first and foremost as an appeal for recognition of his vulnerability:

“*I accept that something in me is broken, but that I can learn to live with that vulnerability (…) Of course I am resilient a well, that's what other people would say of me. But on one point it felt good to acknowledge that, because of events in my youth and afterwards, something inside me is broken, and I won't be the same as before those disruptive experiences.”*

#### Personal Context

John's narrative centers on “where he is coming from and what made him who he is.” He describes how he tackled his childhood detachment and found connectedness with others. Central themes of his narrative are self-insight and self-acceptance. Analyzing his life-history has apparently been an important way of meaning making to John. He starts his telling by linking a childhood of neglect and adaptation to his troubles later in life:

“*I am a child of parents with mental illness. Both ended up in psychiatry. I also have a brother with a birth defect, which impacted our family, and the amount of attention I got. Looking back, I think my parents were not ready to have children. They struggled with their own and troubles. So, in accumulation, these circumstances made me feel very detached. I feel like I have muted my emotions most of my life.”*

We learn that John's struggle with his neglected need to attach and belong becomes the common thread of his life. John stresses his recurrent feelings of being a misfit. For example, when he became the first of his working-class family to go to university: he recalls his great ambitions and his troubles to realize them. Looking back, he now understands his life in terms of self-fulfilling failure: an expectation of not being seen and accepted hindered him to commit to work and relationships in his adult life. John reports how he turned to mental healthcare various times, but felt that the predominant cognitive approaches did not help him to resolve his sense of detachment. Resolving in John's narrative appears after he meets his second spouse after a crisis. She supports him to engage in Mindfulness and peer-support groups, resulting in the insight that only by tuning in to his neglected needs and pain, he can break his pattern of detachment.

#### Ideological Context

As we have seen, John conceptualizes mental distress as a form of detachment that is rooted in his youth. Accordingly, his responsibility in recovery is to stay connected with his feelings and needs, as opposed to “living in his head.” For John, self-connection has become a condition to establish profound relationships with others. John's narrative shows that he appropriated and integrated a wide array of frameworks to give meaning to his struggles. Most prevalent are several psychotherapy frameworks (schema and system therapy) that seem to function primarily as a way to legitimate his difficulties and relate them to his youth. Additionally, a mindfulness framework apparently helped him to find connection to the emotions and bodily dimension of his pain. Within a recovery framework, then, his suffering elevates his status and facilitates a new identity and role as an expert-by-experience.

Although John has a narrative approach to describing his trouble, he also refers to the various diagnostic classifications that apply to him, like CPMI, ADD, depression, dissociation, burn-out etc. This medical terminology seems helpful to him to elicit recognition for his vulnerability and confirm his belonging to a new group. Below he reflects on this process:

“*So instead of thinking, like -what I had in the beginning- do I really fit in? Cause there is this feeling underneath, the need to belong, to connect. Well, I started to realize that what I had gone through in my life was certainly burdensome, and that I carry that load with me. And by recognizing that, I also have a more armory as an expert by experience.”*

#### Conclusion

Through contextual analysis of John's narrative, we learned about the significance for John to be recognized in his vulnerability. In the context of a life-story of neglect and adaptation, he struggled with feelings of detachment from his own needs. He felt unable to live up to the norms of society and belong to a social group. Within a recovery framework, John's personal struggles become a valuable legacy, instead of the failure he once perceived them to be. But above all, an identity as an expert by experience offers him a new, positive identity and becomes an answer to his need to belong.

## Discussion

With this study we wanted to gain contextual insight into personal recovery and recover-support in psychiatry. For this purpose, we inquired 25 narratives of service-users. We focused on three levels of storied context that were offered by the narratives: the personal, interpersonal, and ideological context. Comparative analysis resulted in the identification of four different genres, which we named Lamentation, Reconstruction, Accusation and Travelogue. By connecting the different levels of analysis we gained insight into specific, context-bound difficulties and openings for recovery support.

From Lamentation narratives, we learned that some people may literally need new stories to redefine their “lost selves” amidst mental distress. The desire to return to normality that narrators of this genre express has been identified previously as a theme in a minority of recovery narratives (23). Within our study, this desire, once contextualized is understood as a consequence of intolerance for troubled behavior. In line with previous recovery research, this genre confirms the significance of overcoming stigma (5, 56, 57) and regaining a sense of dignity (58) to develop a new, positive identity after being confronted with mental distress. A contextual approach, however, also showed the difficulty of this process for people that are deprived from the social and narrative resources to move beyond devaluating perceptions of mental distress. Without these resources it becomes harder to mitigate threads of devaluation and protect one's moral status (59). Among others, caretakers^4^ could support people by providing emancipating frameworks of mental distress that are attuned to their social context, and help to restore self-worth.

Despite our transdiagnostic approach we found that one of the genres was exclusively related to (male)experiences of psychosis. Reconstruction narratives demonstrated how the experience of psychosis can complicate the act of meaning making of both past and future life. Meaning making has been identified as a key process of personal recovery and includes making sense of past distress, as well as finding meaning and purpose in life (5, 60, 61). From the context of reconstruction narratives, we learned that past-oriented meaning making can be very threatening for people that were first overwhelmed, and later estranged by the abundance of meaning making during psychotic episodes. This may explain why narrators actively avoid exploration of distressful and traumatic past events. Important to consider here, is that the professionals working with people with psychotic distress, may sustain this pattern of avoidance, due to negative beliefs about their patients' ability to cope with past trauma, (62, 63). Giving meaning to the future was identified as an important struggle in reconstruction narratives too. Although stigma is not explicitly addressed by the narrators, they describe a process of losing previous held hope about possible identities, being replaced by an identity of disability. This “internalized stigma” (64) makes it challenging to envision a satisfying future. In light of the challenges to both past- and future directed meaning making, finding comfort and meaning in the here and now can actually be an adaptive response. However, opening up additional possibilities for meaning-making might further enhance personal recovery. Accumulating service-user based research indicates that making sense of past stress and trauma and integrating these experiences into one's identity and life, are important phases of recovery in psychosis (61). Caretakers thus have an important responsibility to break the mutual circle of fear for deregulation and provide people with the safety and trust needed to facilitate these processes.

Through narratives of accusation, we learned that the dynamics of institutionalized psychiatric practice can form a disempowering and demoralizing experience for people that rely on care. Empowerment is central to conceptualizations of recovery and has been defined in terms of taking responsibility and control over one's own life (5). However, from the context of accusation narratives, we learn that people rely on supportive relationships and systems and develop a sense of control over their own behavior. Tragically, narrators struggle to find such support within psychiatric care. In contrary, they feel that their attempts to take control -by indicating how they understand their troubles and needs in care- is undermined by the power structures and system barriers in institutional psychiatry. Accusation narratives thus highlight that empowerment is not an individual achievement, but a social process that requires equal respect, both within (care)relationships and institutional structures.

In line with previous finding (26), most narrators also identify stigma within psychiatric care as an important hinderer in recovery. They point to the damaging impact on their identity of being reduced to a mental disorder. In accordance with quantitative research findings (65), narratives of accusation illustrate the association between the experience of feeling different from others and feelings of demoralization. In this light, the urgency to be recognized as an equal human being becomes apparent. People who are deeply entangled in (self)stigma depend on supportive relationships to escape the vicious circle of rejection. Caretakers could support this process by overcoming prejudices and fear for troubling behavior and by connecting through equality.

Travelogue narratives illustrated how people can gain a sense of belonging through recognition of their vulnerability. Belonging and connectedness have been defined as a decisive factors in the process of recovery (5, 24). However, whereas previous research has stressed the importance of developing a positive-identity beyond mental distress (60), connectedness in travelogue narratives is established through identification with mental distress. This opposed direction of identity-development can be understood from the context of travelogue narratives. In the light of a history of neglected needs and adaptation, it becomes comprehensible that narrators might first and foremost profit from recognition of their fragility. Contrary to the other genres, narrators of travelogue do not have to disentangle from self-stigma, but rather from ideas about normality. What narratives of travelogue demonstrate, is the importance of space for people to liberate themselves of living up the dominant norms of society, but still be a valued member of it. Caretakers can support this process by dismissing people from the duty to adapt and to help them connect instead to their embodied pain and needs.

## Contributions And Limitations

Researching personal recovery in psychiatry is a challenging enterprise. To develop recovery-oriented care, we need a knowledge base to build on. However, the logics of experience and science are not easily unified. Much qualitative research on personal recovery has focused on mapping and synthesizing shared characteristics and themes of personal accounts in order to gain insight into recovery. However, generalizing the highly unique process of recovery seems a contradictio in terminis. Our narrative approach might help to abate this contradiction by elucidating the shared and differentiating contexts that determine what is of importance to people in recovery, and why. Among others, we found important clues for personal recovery support in people's narrated life-struggles and the interpersonal appeal of their telling. Through contextual analysis, we learned that the recognition that people seek is specific and differentiating. We saw, for example, that recognition of vulnerability and being different can be crucial for people with a life story of adaptation, whereas people that struggle with the stigma of deviation desire to be recognized in their humanity and similarity to others.

The study of narrative is an eligible way to relate the intimate details of personal life stories' with larger paradigms in psychiatry and society (15). However, in mental health research, the balance tends to shift to the detriment of the latter (37). By including the social context of the narratives in our analysis, the dependency of people in recovery on supportive others, care systems and socially available stories was illuminated. Such an approach is important to counterbalance overly individualistic elaborations of recovery, and sustain an emancipatory narrative tradition (66). We demonstrated the value of explicating and connecting different levels of context as proposed by Murray (37) and Zilber, Tuval-Mashiach et al. (29). Our results indicate, for example, that familiarity with a broad repertoire of ideas about mental distress may help people to express and reframe their personal struggles and desires in a helpful way, thereby facilitating interpersonal recognition.

The variety of narrative genre within a small sample implicates that many different stories about mental distress and recovery are possible. This might be particularly important in the light of critiques on narrative normativity. With regard to the content of recovery stories, Fisher and Lees (66) have argued that current mental health approaches might impose narratives of individual achievement and autonomy to people, to the expanse of relational ways of envisioning recovery. Additionally, expectations about recovery as a linear process can increase marginalization and a sense of failure when people do not meet normative milestones (66, 67). With regard to form, dominant cultural expectations of “well formed narratives” -such as coherence and temporal ordering (68, 69), and their relation to wellbeing (70) have been challenged as well. Consequently, various authors have argued that in order to preserve an emancipatory narrative tradition in mental health, extension of available narrative templates is necessary (43, 44, 66, 71). Our results might offer such extension, and indicate that different narrative genres, comprise different qualities. While Travelogue for instance, might be the “ideal” recovery story of insight and inspiration, the indignation of narratives of accusation might be needed to provoke social change. Additionally, Lamentation and Reconstruction demonstrate that narratives with less articulated plots and coherence still offer abundant insight into people's lifeworld's. Thus, creating space for narrative plurality, also requires listeners to reflect on their own narrative preferences and discomforts (17). Some methodological limitations to our work are worth noting. Firstly, our attempt to cover and relate different contexts of narratives resulted in an pluralistic analytical framework. Although “methological pluralism” may be needed to capture the richness of narrative data and promote integrated theory development (46), it can also pose a threat to theoretical coherence (72). Secondly, it is important to acknowledge that our findings are grounded in one-time interviews that give a momentary impression of the evolving process of meaning making that personal recovery entails. The narratives people construct in a research setting are like a “frozen, still photograph” of their dynamically changing perceptions of their selves and lives [(30), 8]. Although we showed that even one-time interviews offer insight into the dynamics of meaning-making, as people self-report on important changes in these perceptions, future narrative research with a longitudinal design could provide deeper insight into how meaning-making is transformed over time, and under what circumstances. Thirdly, our initial recruitment strategy led to selection bias. The majority of our participants were middle-aged, native Dutch people with a high level of education. We noted that online recruitment was more likely to attract people that were empowered in terms of their social and intellectual resources. As we started to actively reach out for socially disadvantaged groups, we learned that our recruitment strategies had a direct impact on narrative diversity and the genres we were able to identify. For example, the genre of lamentation, centered around social (status) loss, was solely represented by people recruited in community centers in deprived neighborhoods. We therefore endorse the plea of Karadzhov (73) for better documentation and contextualization of socio-demographic characteristics in recovery research, in order to assess the impact of social inequalities on the recovery process. Lastly, qualitative studies such as these are not intended, nor suitable for generalization to the population (statistical generalization). Instead, theoretical, or conceptual generalization applies (74).

In this paper we demonstrated the importance of a contextual understanding of recovery. We argued that regard for context in recovery research is an important means to make context matter in psychiatric practice. We believe that most caretakers in psychiatry share a deep interest in the stories and context of their individual patients. However, with the establishment of the DSM as the organizing principle in psychiatry (47), clinical conversation has been increasingly limited to the assessment of specific conditions, thereby creating a form of “institutionalized tunnel vision” [(75), 372]. Understanding emotional distress and troubled behavior exclusively in terms of psychiatric symptoms tends to obscure the meaning-based links between adverse life circumstances, power inequity, and peoples intelligible responses to them (18). Both the methods and findings of narrative research have the potential to restore these links and open up clinical conversation. Based on the current study, we encourage caretakers to listen for personal life struggles, beyond topical symptoms; for the appeal of their patients' story, beyond the explicit request for help; and for the ideological embedding of the personal story. Listening this way may provide deeper insight into the ways people want to be recognized and offer openings to support them in granting that recognition.

## Data Availability Statement

The datasets presented in this article are not readily available because of participant confidentiality and privacy. Requests to access the datasets should be directed to Nienke van Sambeek, n.vansambeek@umcutrecht.nl.

## Ethics Statement

Ethical review and approval was not required for the study on human participants in accordance with the local legislation and institutional requirements. The patients/participants provided their written informed consent to participate in this study.

## Author Contributions

FS, GF, and SG participated in conception, design and acquisition of the Psychiatry Story Bank project. Methodology design and data-analysis of the study was performed by NS and supervised by AB. GF and FS validated the outcomes of the analysis. NS, AB, GF, SG, and FS contributed to interpretation of the data. The first draft of the manuscript was written by NS. All authors contributed to manuscript revision, read, and approved the submitted version.

## Funding

This study was funded by Foundation VCVGZ (project number 254) that finances innovative projects in mental health care.

## Conflict of Interest

The authors declare that the research was conducted in the absence of any commercial or financial relationships that could be construed as a potential conflict of interest.

## Publisher's Note

All claims expressed in this article are solely those of the authors and do not necessarily represent those of their affiliated organizations, or those of the publisher, the editors and the reviewers. Any product that may be evaluated in this article, or claim that may be made by its manufacturer, is not guaranteed or endorsed by the publisher.

## Footnotes

^1^These processes were identified in a systematic review and narrative synthesis of 97 papers, and were given the acronym CHIME. Although there is ongoing debate about definitions of personal recovery, the CHIME framework continues to be a widely endorsed conceptual framework, see (6).

^2^ https://psychiatrieverhalenbank.nl/

^3^In his initial proposal (37),distinguishes between four levels of analysis, including a “positional” level that entails analysis of the differences in social position between the interviewer and respondent. Although the positional level was discussed in reflection sessions, we decided to exclude it from analysis because our data (collection) did not allow for thorough analysis of this kind.

^4^In line with the aim of our research- we focused here on the ways caretakers in psychiatry can support recovery. It is important to note, however, that we do not assume that all difficulties are best addressed within psychiatry. Many difficulties, especially that of stigma, ask for peer-support, community work and public debate to enhance changes in the way we perceive and deal with mental distress in society.
