# Supplementary material for: Recovering Context in Psychiatry: What Contextual Analysis of Service Users' Narratives Can Teach About Recovery Support
Source: Front Psychiatry. 2021 Dec 20;12:773856. doi: 10.3389/fpsyt.2021.773856 (PMC8720875; doi:10.3389/fpsyt.2021.773856)
Supplement: Supplementary file 1 [file Data_sheet_1.docx]

**Appendix I: Interview guide**

| **Main story and meaning** | |
| --- | --- |
| Opening question: What story would you like to share with us?  Alternative question: Could you tell what motivated you to share your story? | |
| **Topics to inquire**  *(to inquire when not brought up spontaneously)* | **Example questions**  *(to be adjusted to evolving story)* |
| Identity | How did these experiences affect the way you perceive yourself? |
| Participation | How did these experiences affect your work/activities?  How did these experiences affect your position in society? |
| Connectedness | How did people react to these experiences?  Did you experience support from your loved-ones? |
| Care | What role did mental health care play in these experiences?  What helped or hindered in care? |
| Meaning | How do you look back on these experiences?  How do you look at your future after these experiences? |

**Appendix II: Explanation of analyzed characteristics**

|  | Characteristic | *Question* | *Operationalization* | *Sources* |
| --- | --- | --- | --- | --- |

| Personal level | Subject | What is the narrative mainly about? | a) Categorization of the text into overarching subjects.  b) Computer assisted determination of the subject most of the text refers to (coverage was queried with Nvivo 12 software ). | Inspired by elaborations of recovery narratives as one possible, but narrow genre (Woods, Hart, & Spandler, 2019), to be distinguished from illness narratives (Llewellyn-Beardsley et al., 2019). |
| --- | --- | --- | --- | --- |
|  | **Themes** | What are the central issues brought forward in the narrative? | Systematic analysis of themes: summaries of what is going on in each fragment.  Coding was performed inductively, on a semantic level and performed with NVivo 12 software. | Based on guidelines for thematic analysis from Braun and Clarke (2006) and Rubin and Rubin (2005).  Inspired by the elaboration of Johnstone and Boyle (2018) of narrative as a means to restore link between adverse life experiences and people’s intelligible responses to them. |
|  | **Life story** | How can the narrated life-story be characterized? | Characterization through close-reading of references to (adverse) childhood, life-events and recurrent patterns of interaction in the past. |  |
|  | **Struggles** | Which personal struggles are expressed in de narrative? | Systematic analysis of struggles: all references to urgent, personal difficulties that cause worry and require effort. Reformulated as ‘I have to…’ | Based on the example of Olthuis et al. (2014) and inspired by studies that identified ‘ongoing struggles’ (De Smet et al., 2020) and ‘difficulties’ (Stuart, Tansey, & Quayle, 2017; van Weeghel, van Zelst, Boertien, & Hasson-Ohayon, 2019) as an underexposed aspect of recovery from mental distress. |
|  | **Resolving** | How is the core-struggle overcome or dealt with? | Assessment of:  -Helpers: factors that positively contribute to overcoming or dealing with specific struggles.  -Turning points: References to crucial, specific experience(s) that mark the move to a different trajectory in recovery or gradual processes of change. | Inspired by the work of Dumont et al. (2006), Onken et al. (2002) and (Kogstad, Ekeland, & Hummelvoll, 2011)  that demonstrated the value of identifying helping factors and turning points to identify openings for recovery support. Adapted to our contextual approach by relating them to struggles. |
| Interpersonal level | **Purpose** | What is the purpose of the telling? | Assessment of:  -The narrators’ explicit reference to his/her motivation to tell.  -Implicit cues in the interaction or style of telling. | Inspired by elaborations of narrative as an intentional, performative act with a social role (Atkinson & Delamont, 2006; Bamberg, 1997; Murray & Sools, 2015; Riessman, 2008). |
|  | **Audience** | Who is the intended recipient of the telling? | Assessment of all (in)direct reference to the intended audience the narrator wants to reach. |  |
|  | **Emotional tone** | What is the prevailing expressed emotion in the telling? | Assessment of intonation (and/or transcribed intonation cues). | Literature: Based on Llewellyn-Beardsley et al. (2019) and Anderson and Kirkpatrick (2016) that identified tone as an important narrative characteristic. |
|  | **Structuring** | How is the telling structured? | Characterization through close-reading of the way the narrator connects different experiences to each other. | Inspired by elaborations on the narrative structure of experience, plot as a structuring device and differences in coherence (Frank, 1995; Llewellyn-Beardsley et al., 2019; Mattingly, 1998; Westerhof & Bohlmeijer, 2012). |
|  | **Appeal** | How does the narrator want to be recognized by the audience? | Assessment of:  - (In)direct-reference how someone wants to be seen/characteristics that narrator highlights of him/herself.  -Reference to interactions in which people felt particularly (un)seen/(un)heard. | Inspired by the notion of recovery/illness narratives as a form of recognition seeking (Fisher, 2008; Frank, 1995) and Honneth’s theorization of (modes of) recognition as a preconditions for self-realization, and social conflict as motivated by the experience of being denied these conditions (Honneth, 1996). |
| Ideological level | **MI construction** | How does the narrator construct his/her understanding of mental distress in language? | Systematic analysis of all statements, terminology, and metaphors in talk about mental distress. | Based on the concept of ‘discursive constructions’* from (critical) discourse analysis, as described by Ussher and Perz (2014), Van Dijk (2015) and Montessori, Schuman, and Lange (2012). |
|  | **MI framework** | Which framework(s) of mental distress are dominant in the narrative? | Comparison of identified metaphor, statements and terminology with those of known frameworks of mental distress.  Distinctions of different models and metaphors of mental distress by Lewis (2011) were used to help identify frameworks. | Based on conceptualizations of narrative as embedded in socially available ‘meta-narratives’ (Czarniawska, 2004; Kirkpatrick, 2008; Shapiro, 2011; Spector-Mersel & Knaifel, 2018) or discourses (Jorgensen & Phillips, 2002). |
|  | **Related identity** | Which identity is made possible by the narrator’s preferential framework of mental distress? | Systematic analysis of:  a) the words people use to refer to themselves  b) We/they references to identify inside/outside group and the characteristics attributed to them. | Based on the discourse analytical concept of ‘subject positioning’ that clarifies the link between discourse and possible identities, as described by Ussher and Perz (2014) and Törrönen (2001). Informed by Harper (1995) and Speed (2006). |
|  | **Related function** | What is gained by using this framework? | Assessment of positive feelings- or actions that are enhanced by the framework. | Based on the discourse analytical idea that people can gain something by making use of specific discourses, see example of Ussher and Perz (2014). |
|  | **Related responsibility**  **in recovery** | What are the consequences of the framework in terms of actions to be undertaken for recovery? | Assessment of references to duties or goals in recovery. | Drawing on the example of discourse related practices, as described by Ussher and Perz (2014) Inspired by the work of Frank (1995, 2012) and Lewis (2011) that highlights the ethical dimension of patient narratives and clinical models, demonstrating that each framework has different real life consequences. |

**References**

Anderson, C., & Kirkpatrick, S. (2016). Narrative interviewing. *International Journal of Clinical Pharmacy, 38*(3), 631-634. doi:10.1007/s11096-015-0222-0

Atkinson, P., & Delamont, S. (2006). Rescuing narrative from qualitative research. *Narrative Inquiry, 16*(1), 164-172.

Bamberg, M. G. W. (1997). Positioning between structure and performance. *Journal of Narrative & Life History, 7*(1-4), 335-342. doi:10.1075/jnlh.7.42pos

Braun, V., & Clarke, V. (2006). Using thematic analysis in psychology. *Qualitative Research in Psychology, 3*(2), 77-101.

Czarniawska, B. (2004). *Narratives in Social Science Research*: SAGE publications.

De Smet, M. M., Meganck, R., De Geest, R., Norman, U. A., Truijens, F., & Desmet, M. (2020). What "good outcome" means to patients: Understanding recovery and improvement in psychotherapy for major depression from a mixed-methods perspective. *Journal of counseling psychology, 67*(1), 25-39. doi:10.1037/cou0000362

Fisher, P. (2008). Wellbeing and empowerment: the importance of recognition. *Sociology of Health & Illness, 30*(4), 583-598. doi:10.1111/j.1467-9566.2007.01074.x

Frank, A. W. (1995). *The Wounded Storyteller: Body, Illness, and Ethics* (2nd ed.): University of Chicago Press.

Frank, A. W. (2012). *Letting Stories Breathe: A Socio-Narratology* University of Chicago Press.

Harper, D. J. (1995). Discourse analysis and 'mental health'. *Journal of Mental Health, 4*(4), 347-358. doi:10.1080/09638239550037406

Honneth, A. (1996). *The Struggle for Recognition: The Moral Grammar of Social Conflicts*: Polity Press.

Johnstone, L., & Boyle, M. (2018). The Power Threat Meaning Framework: An Alternative Nondiagnostic Conceptual System. *Journal of Humanistic Psychology, 0*(0), 0022167818793289. doi:10.1177/0022167818793289

Jorgensen, M., & Phillips, L. (2002). *Discourse Analysis as Theory and Method*. London: SAGE Publications.

Kirkpatrick, H. (2008). A narrative framework for understanding experiences of people with severe mental illnesses. *Arch Psychiatr Nurs, 22*(2), 61-68. doi:10.1016/j.apnu.2007.12.002

Kogstad, R. E., Ekeland, T.-J., & Hummelvoll, J. K. (2011). In defence of a humanistic approach to mental health care: recovery processes investigated with the help of clients' narratives on turning points and processes of gradual change. *18*(6), 479-486. doi:10.1111/j.1365-2850.2011.01695.x

Lewis, B. (2011). *Narrative psychiatry: How stories can shape clinical practice*. Baltimore, MD, US: Johns Hopkins University Press.

Llewellyn-Beardsley, J., Rennick-Egglestone, S., Callard, F., Crawford, P., Farkas, M., Hui, A., . . . Slade, M. (2019). Characteristics of mental health recovery narratives: Systematic review and narrative synthesis. *PLOS ONE, 14*(3), e0214678. doi:10.1371/journal.pone.0214678

Mattingly, C. (1998). *Healing dramas and clinical plots: The narrative structure of experience* (Vol. 7): Cambridge University Press.

Montessori, N. M., Schuman, H., & Lange, R. (2012). *Kritische discoursanalyse: De macht en kracht van taal en tekst*: Academic and Scientific Publishers

Murray, M., & Sools, A. (2015). Narrative research. In P. Rohleder & A. C. Lyons (Eds.), *Qualitative research in Clinical and Health Psychology* London: Palgrave.

Olthuis, G., Prins, C., Smits, M.-J., van de Pas, H., Bierens, J., & Baart, A. (2014). Matters of Concern: A Qualitative Study of Emergency Care From the Perspective of Patients. *Annals of Emergency Medicine, 63*(3), 311-319.e312. doi:<https://doi.org/10.1016/j.annemergmed.2013.08.018>

Riessman, C. K. (2008). *Narrative methods for the human sciences*: Sage publications.

Rubin, H. J., & Rubin, I. S. (2005). *Qualitative Interviewing: The Art of Hearing Data* (2nd ed.). California: Thousand Oaks.

Shapiro, J. (2011). Illness narratives: reliability, authenticity and the empathic witness. *Medical Humanities, 37*(2), 68-72. doi:10.1136/jmh.2011.007328

Spector-Mersel, G., & Knaifel, E. (2018). Narrative research on mental health recovery: two sister paradigms. *Journal of Mental Health, 27*(4), 298-306. doi:10.1080/09638237.2017.1340607

Speed, E. (2006). Patients, consumers and survivors: A case study of mental health service user discourses. *Social Science & Medicine, 62*(1), 28-38. doi:<https://doi.org/10.1016/j.socscimed.2005.05.025>

Stuart, S., Tansey, L., & Quayle, E. (2017). What we talk about when we talk about recovery: a systematic review and best-fit framework synthesis of qualitative literature. *Journal of Mental Health, 26*, 1-14. doi:10.1080/09638237.2016.1222056

Törrönen, J. (2001). The Concept of Subject Position in Empirical Social Research. *Journal for the Theory of Social Behaviour, 31*, 313-330. doi:10.1111/1468-5914.00161

Ussher, J., & Perz, J. (2014). Discourse Analysis. In P. Rohleder & A. C. Lyons (Eds.), *Qualitative Research in Clinical and Health Psychology* (pp. 218-237): Palgrave MacMillan.

Van Dijk, T. A. (2015). Critical Discourse Analysis. In D. Tannen, H. E. Hamilton, & D. Schiffrin (Eds.), *The Handbook of Discourse Analysis* (pp. 466-485).

van Weeghel, J., van Zelst, C., Boertien, D., & Hasson-Ohayon, I. (2019). Conceptualizations, assessments, and implications of personal recovery in mental illness: A scoping review of systematic reviews and meta-analyses. *Psychiatric Rehabilitation Journal, 42*(2), 169-181. doi:10.1037/prj0000356

Westerhof, G. J., & Bohlmeijer, E. T. (2012). Life Stories and Mental Health: The Role of Identification Processes in Theory and Interventions. *Narrative Works, 2*(1). Retrieved from <https://journals.lib.unb.ca/index.php/NW/article/view/19501>

Woods, A., Hart, A., & Spandler, H. (2019). The recovery narrative : politics and possibilities of a genre. *Culture, medicine, and psychiatry.* Retrieved from <http://dro.dur.ac.uk/27751/>
